# (3a*R*,4*S*,7*R*,7a*S*)-2-Phenyl-4-propyl-3a,4,7,7a-tetra­hydro-1*H*-4,7-epithio­iso­indole-1,3-dione 8-oxide

**DOI:** 10.1107/S1600536811012876

**Published:** 2011-04-13

**Authors:** Aydın Demircan, Ertan Şahin, Gözde Beyazova, Muhsin Karaaslan, Tuncer Hökelek

**Affiliations:** a Department of Chemistry, Niğde University, 51100 Niğde, Turkey; b Department of Chemistry, Atatürk University, 25240 Erzurum, Turkey; c Department of Chemistry, Aksaray University, 68100 Aksaray, Turkey; d Department of Physics, Hacettepe University, 06800 Beytepe, Ankara, Turkey

## Abstract

In the tetra­hydro­isoindole moiety of the title compound, C_17_H_17_NO_3_S, the six-membered ring assumes a boat configuration and the –S=O group bridges the prow and stern of the boat. The phenyl ring is oriented at a dihedral angle of 83.2 (1)° with respect to the pyrrole ring. In the crystal, inter­molecular C—H⋯O hydrogen bonds link the mol­ecules into a three-dimensional network. A weak C—H⋯π inter­action involving the phenyl ring is also found. The crystal studied was an inversion twin.

## Related literature

For background to the thio­phenen system, see: Lert & Trindle (1971 ▶). For the conditions of cyclo­addition reactions of thio­phene, see: Al-Omran *et al.* (1996 ▶); Kuhn & Gollnick (1972 ▶); Kotsuki *et al.* (1978 ▶); Thiemann *et al.* (1995 ▶). For the biological activity of some thio­phene 1,1-dioxide derivatives, see: Thiemann *et al.* (2009 ▶). For thio­phene *s*-oxides with alkyl groups at positions 2,3,4 and 5, see: Rajappa (1984 ▶). For related structures, see: Arslan & Demircan (2007 ▶); Koşar *et al.* (2006 ▶).
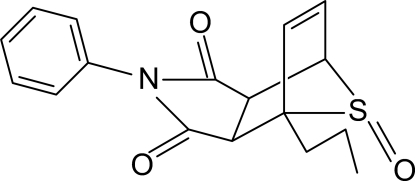


## Experimental

### 

#### Crystal data

C_17_H_17_NO_3_S
*M* *_r_* = 315.39
Orthorhombic, 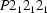

*a* = 7.7712 (3) Å
*b* = 10.8413 (3) Å
*c* = 18.9762 (4) Å
*V* = 1598.74 (8) Å^3^
*Z* = 4
Mo *K*α radiation
μ = 0.21 mm^−1^
*T* = 294 K
0.30 × 0.25 × 0.20 mm

#### Data collection

Rigaku R-AXIS RAPID-S diffractometer
Absorption correction: multi-scan (Blessing, 1995 ▶) *T*_min_ = 0.807, *T*_max_ = 0.865
34450 measured reflections
3279 independent reflections
2686 reflections with *I* > 2σ(*I*)
*R*_int_ = 0.088

#### Refinement

*R*[*F* ^2^ > 2σ(*F* ^2^)] = 0.047
*wR*(*F* ^2^) = 0.108
*S* = 1.08
3279 reflections
210 parameters
H atoms treated by a mixture of independent and constrained refinement
Δρ_max_ = 0.20 e Å^−3^
Δρ_min_ = −0.22 e Å^−3^
Absolute structure: Flack (1983 ▶), 1379 Friedel pairs
Flack parameter: 0.37 (9)

### 

Data collection: *CrystalClear* (Rigaku/MSC, 2005 ▶); cell refinement: *CrystalClear*; data reduction: *CrystalClear*; program(s) used to solve structure: *SHELXS97* (Sheldrick, 2008 ▶); program(s) used to refine structure: *SHELXL97* (Sheldrick, 2008 ▶); molecular graphics: *ORTEP-3 for Windows* (Farrugia, 1997 ▶); software used to prepare material for publication: *WinGX* (Farrugia, 1999 ▶) and *PLATON* (Spek, 2009 ▶).

## Supplementary Material

Crystal structure: contains datablocks I, global. DOI: 10.1107/S1600536811012876/xu5179sup1.cif

Structure factors: contains datablocks I. DOI: 10.1107/S1600536811012876/xu5179Isup2.hkl

Additional supplementary materials:  crystallographic information; 3D view; checkCIF report

## Figures and Tables

**Table 1:** Hydrogen-bond geometry (Å, °) *Cg*1 is the centroid of the C12–C17 phenyl ring.

*D*—H⋯*A*	*D*—H	H⋯*A*	*D*⋯*A*	*D*—H⋯*A*
C5—H5⋯O1^i^	0.95 (2)	2.51 (3)	3.326 (4)	144 (2)
C17—H17⋯O3^ii^	0.93	2.57	3.315 (3)	137
C7—H7⋯*Cg*1^iii^	0.98	2.77	3.693 (3)	157

Symmetry codes: (i) 
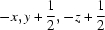
; (ii) 
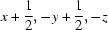
; (iii) 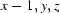.

**Fig. 1.**
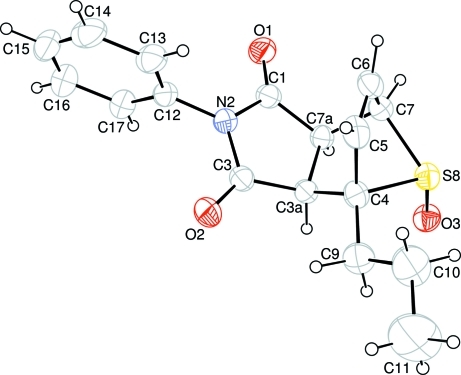
The molecular structure of the title molecule with the atom-numbering scheme. Displacement ellipsoids are drawn at the 40% probability level.

**Fig. 2.**
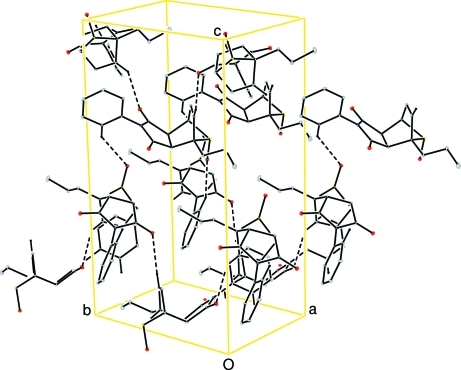
A view of the crystal packing of the title compound. The C-H···O hydrogen bonds are shown as dashed lines [H-atoms not involved in hydrogen bonding have been omitted for clarity].

## supplementary crystallographic information

### Comment

Thiophene behaves as the least reactive diene among all aromatic five-membered heterocycles; the presence of 3d orbitals on sulfur contributes to the resonance stability of the thiophene system (Lert & Trindle, 1971). Cycloaddition reactions of thiophene also have limitations therefore its 4 + 2 cycloaddition can occur under following conditions; use of highly reactive dienophiles (Thiemann *et al.*, 1995), enhance electron density on the thiophene by having substituent at position 2 or 5 (Al-Omran *et al.*, 1996), high reaction temperature (Kuhn & Gollnick, 1972) or use of high pressure (Kotsuki  *et al.*, 1978). However, the number of publications dealing with the chemistry of thiophene dioxide based new materials, namely, semiconductor organic transistors and light diodes first designed about 13 years ago. New data on the biological activities of some thiophene 1,1-dioxide derivatives were observed in recent years (Thiemann *et al.*, 2009).

Thiophene *s*-oxides are derivatives of thiophene, which belong to an important group of five-membered heterocyclic compounds having non-aromatic character. Thiophene *s*-oxides with alkyl groups at positions 2,3,4 and 5 have been the subject of extensive studies in recent past (Rajappa, 1984). Owing to the unique structure combined with high reactivity, they can be used to prepare various heterocyclic systems; therefore, these compounds are useful building blocks in synthetic organic chemistry. In continuation of our research program and following our previous interest in the sytheses of fused heterocyclic compounds using furan (Arslan & Demircan, 2007; Koşar *et al.*, 2006), it has been found that mono alkylated thiophene at position 2 will lead to an excellent building block for the synthesis of the title compound.

The title compound contains two non-planar five- and six-membered rings, which has a pyrrole (C1/N2/C3/C3a/C7a) ring (B) on one side and a propyl moiety at position 4. It also contains a phenyl (C12-C17) ring (A) bonded to the pyrrole ring (B) at position 2. The angles C4-C5-C6 [111.4 (3)°] and C5-C6-C7 [111.1 (3)°] about the double bond have an average value of 111.3 (3)°. The dihedral angles between planes A, B, C (C3a/C4/C7a C7), D (C4-C7) and E (C4/S8/C7) are as follows: A/B = 83.2 (1)°, B/C = 61.9 (1)°, C/D = 60.6 (1)°, C/E = 58.8 (1)° and D/E = 60.7 (1)°.

In the crystal, intermolecular C—H···O hydrogen bonds link the molecules to form a three-dimensional network (Table 1 and Fig. 2). There also exist a weak C—H···π interaction involving the phenyl ring A (C12-C17) (Table 1).

### Experimental

For the preparation of the title compound, BF_3_.Et_2_O (6.1 ml, 47.75 mmol) was added slowly to a solution of 2-propylthiophene (1.00 g, 7.90 mmol) and N-phenylmaleimide (2.06 g, 11.90 mmol) in dry dichloromethane (DCM) (40 ml) under an inert atmosphere at 253 K. The reaction mixture was stirred for 10 min at 253 K, and then a solution of meta chloroperbenzoic acid (*m*-CPBA) (2.05 g, 11.90 mmol) in dry DCM (40 ml) was added slowly. The reaction mixture was stirred for 2 h at 253 K, and then the suspension was poured into a mixture of aqueous NaHCO_3_ solution (80 ml) and DCM (50 ml) and stirred at room temperature for 20 min. The organic phase was separated, and the aqueous phase was extracted with DCM (3 x 25 ml). The combined organic phase was washed with water and brine, and then dried over anhydrous MgSO_4_. After removal of the solvent *in vacuo*, the residue was chromatographed on silica gel to give the title compound as colorless crystals.

### Refinement

The H atoms H5 and H6 were located in a difference Fourier map and were freely refined. Other C-bound H-atoms were positioned geometrically with C—H = 0.93, 0.96, 0.97 and 0.98 Å, for aromatic, methyl, methylene and methine H-atoms, respectively, and constrained to ride on their parent atoms, with *U*_iso_(H) = k × *U*_eq_(C), where k = 1.5 for methyl H-atoms and k = 1.2 for all other H-atoms.

### Crystal data

**Table:**

C_17_H_17_NO_3_S	*F*(000) = 664
*M**_r_* = 315.39	*D*_x_ = 1.310 Mg m^−^^3^
Orthorhombic, *P*2_1_2_1_2_1_	Mo *K*α radiation, λ = 0.71073 Å
Hall symbol: P 2ac 2ab	Cell parameters from 6907 reflections
*a* = 7.7712 (3) Å	θ = 2.2–26.4°
*b* = 10.8413 (3) Å	µ = 0.21 mm^−^^1^
*c* = 18.9762 (4) Å	*T* = 294 K
*V* = 1598.74 (8) Å^3^	Block, colorless
*Z* = 4	0.30 × 0.25 × 0.20 mm

### Data collection

**Table:**

Rigaku R-AXIS RAPID-S diffractometer	3279 independent reflections
Radiation source: fine-focus sealed tube	2686 reflections with *I* > 2σ(*I*)
graphite	*R*_int_ = 0.088
ω scans	θ_max_ = 26.4°, θ_min_ = 2.2°
Absorption correction: multi-scan (Blessing, 1995)	*h* = −9→9
*T*_min_ = 0.807, *T*_max_ = 0.865	*k* = −13→13
34450 measured reflections	*l* = −23→23

### Refinement

**Table:**

Refinement on *F*^2^	Hydrogen site location: inferred from neighbouring sites
Least-squares matrix: full	H atoms treated by a mixture of independent and constrained refinement
*R*[*F*^2^ > 2σ(*F*^2^)] = 0.047	*w* = 1/[σ^2^(*F*_o_^2^) + (0.0453*P*)^2^ + 0.1086*P*] where *P* = (*F*_o_^2^ + 2*F*_c_^2^)/3
*wR*(*F*^2^) = 0.108	(Δ/σ)_max_ < 0.001
*S* = 1.08	Δρ_max_ = 0.20 e Å^−^^3^
3279 reflections	Δρ_min_ = −0.22 e Å^−^^3^
210 parameters	Extinction correction: *SHELXL97* (Sheldrick, 2008), Fc^*^=kFc[1+0.001xFc^2^λ^3^/sin(2θ)]^-1/4^
0 restraints	Extinction coefficient: 0.018 (2)
Primary atom site location: structure-invariant direct methods	Absolute structure: Flack (1983), 1379 Friedel pairs
Secondary atom site location: difference Fourier map	Flack parameter: 0.37 (9)

### Special details

**Table:**

Geometry. All esds (except the esd in the dihedral angle between two l.s. planes) are estimated using the full covariance matrix. The cell esds are taken into account individually in the estimation of esds in distances, angles and torsion angles; correlations between esds in cell parameters are only used when they are defined by crystal symmetry. An approximate (isotropic) treatment of cell esds is used for estimating esds involving l.s. planes.
Refinement. Refinement of F^2^ against ALL reflections. The weighted R-factor wR and goodness of fit S are based on F^2^, conventional R-factors R are based on F, with F set to zero for negative F^2^. The threshold expression of F^2^ > 2sigma(F^2^) is used only for calculating R-factors(gt) etc. and is not relevant to the choice of reflections for refinement. R-factors based on F^2^ are statistically about twice as large as those based on F, and R- factors based on ALL data will be even larger.

### Fractional atomic coordinates and isotropic or equivalent isotropic displacement parameters (Å^2^)

**Table:**

	*x*	*y*	*z*	*U*_iso_*/*U*_eq_	
O1	−0.0424 (3)	0.0571 (2)	0.16742 (11)	0.0742 (6)	
O2	0.3289 (2)	0.35385 (18)	0.09320 (10)	0.0708 (6)	
O3	−0.3050 (2)	0.40176 (16)	0.00144 (9)	0.0633 (5)	
C1	0.0032 (3)	0.1488 (3)	0.13751 (14)	0.0553 (6)	
N2	0.1690 (3)	0.1987 (2)	0.14224 (10)	0.0528 (5)	
C3	0.1923 (3)	0.3011 (2)	0.10005 (12)	0.0526 (6)	
C3A	0.0230 (3)	0.3335 (2)	0.06695 (13)	0.0488 (6)	
H3A	0.0324	0.3349	0.0155	0.059*	
C4	−0.0495 (3)	0.4576 (2)	0.09505 (13)	0.0541 (6)	
C5	−0.0650 (4)	0.4517 (3)	0.17396 (15)	0.0654 (8)	
H5	−0.003 (3)	0.503 (2)	0.2053 (14)	0.060 (8)*	
C6	−0.1750 (4)	0.3652 (3)	0.19369 (15)	0.0659 (8)	
H6	−0.216 (4)	0.346 (3)	0.2393 (16)	0.083 (9)*	
C7	−0.2475 (3)	0.2998 (2)	0.13186 (13)	0.0578 (7)	
H7	−0.3499	0.2498	0.1414	0.069*	
C7A	−0.1015 (3)	0.2327 (2)	0.09100 (12)	0.0509 (6)	
H7A	−0.1479	0.1875	0.0505	0.061*	
S8	−0.28413 (8)	0.43745 (6)	0.07643 (3)	0.0564 (2)	
C9	0.0304 (4)	0.5709 (3)	0.06204 (17)	0.0716 (8)	
H9A	0.1537	0.5675	0.0698	0.086*	
H9B	0.0113	0.5671	0.0116	0.086*	
C10	−0.0343 (5)	0.6940 (3)	0.0883 (2)	0.1081 (13)	
H10A	−0.1584	0.6967	0.0831	0.130*	
H10B	−0.0081	0.7014	0.1381	0.130*	
C11	0.0429 (8)	0.8016 (4)	0.0500 (3)	0.176 (3)	
H11A	−0.0063	0.8768	0.0675	0.264*	
H11B	0.0192	0.7943	0.0005	0.264*	
H11C	0.1650	0.8026	0.0574	0.264*	
C12	0.3072 (3)	0.1422 (2)	0.18063 (14)	0.0580 (7)	
C13	0.3496 (4)	0.1844 (3)	0.24562 (15)	0.0729 (9)	
H13	0.2888	0.2488	0.2663	0.087*	
C14	0.4881 (5)	0.1281 (4)	0.2808 (2)	0.0951 (12)	
H14	0.5197	0.1554	0.3255	0.114*	
C15	0.5753 (5)	0.0350 (4)	0.2504 (3)	0.1005 (14)	
H15	0.6669	−0.0010	0.2743	0.121*	
C16	0.5321 (5)	−0.0070 (4)	0.1856 (2)	0.0974 (12)	
H16	0.5939	−0.0710	0.1651	0.117*	
C17	0.3949 (4)	0.0460 (3)	0.15000 (16)	0.0732 (8)	
H17	0.3626	0.0168	0.1059	0.088*	

### Atomic displacement parameters (Å^2^)

**Table:**

	*U*^11^	*U*^22^	*U*^33^	*U*^12^	*U*^13^	*U*^23^
O1	0.0578 (11)	0.0808 (14)	0.0841 (14)	−0.0097 (11)	−0.0041 (10)	0.0270 (12)
O2	0.0467 (10)	0.0775 (13)	0.0883 (14)	−0.0098 (9)	0.0005 (9)	0.0129 (11)
O3	0.0654 (12)	0.0712 (12)	0.0533 (10)	0.0027 (10)	−0.0088 (9)	−0.0030 (9)
C1	0.0522 (15)	0.0627 (17)	0.0509 (14)	−0.0031 (12)	0.0019 (12)	0.0021 (13)
N2	0.0457 (11)	0.0591 (12)	0.0537 (12)	−0.0010 (9)	−0.0012 (9)	0.0057 (10)
C3	0.0486 (15)	0.0587 (14)	0.0506 (13)	0.0003 (13)	0.0024 (11)	−0.0015 (11)
C3A	0.0463 (13)	0.0526 (14)	0.0476 (13)	0.0001 (10)	0.0013 (11)	0.0006 (11)
C4	0.0499 (14)	0.0552 (15)	0.0573 (15)	−0.0011 (12)	0.0010 (12)	−0.0058 (12)
C5	0.0577 (16)	0.081 (2)	0.0572 (16)	0.0093 (16)	−0.0064 (14)	−0.0204 (15)
C6	0.0559 (17)	0.090 (2)	0.0521 (16)	0.0140 (16)	0.0059 (13)	−0.0032 (15)
C7	0.0482 (15)	0.0675 (16)	0.0578 (15)	−0.0004 (12)	0.0037 (12)	0.0069 (13)
C7A	0.0458 (13)	0.0571 (14)	0.0499 (14)	−0.0020 (11)	−0.0026 (11)	0.0012 (11)
S8	0.0511 (4)	0.0604 (4)	0.0576 (4)	0.0037 (3)	−0.0014 (3)	−0.0029 (3)
C9	0.0703 (18)	0.0600 (17)	0.084 (2)	−0.0091 (15)	0.0010 (15)	−0.0013 (16)
C10	0.111 (3)	0.0577 (19)	0.156 (4)	−0.0059 (19)	0.021 (3)	−0.007 (2)
C11	0.196 (6)	0.061 (3)	0.272 (7)	−0.023 (3)	0.070 (5)	0.004 (3)
C12	0.0467 (14)	0.0694 (17)	0.0579 (15)	−0.0037 (13)	−0.0035 (12)	0.0137 (13)
C13	0.0675 (18)	0.090 (2)	0.0611 (17)	−0.0115 (17)	−0.0119 (14)	0.0085 (15)
C14	0.081 (3)	0.129 (3)	0.075 (2)	−0.035 (2)	−0.026 (2)	0.033 (2)
C15	0.062 (2)	0.127 (4)	0.112 (3)	0.000 (2)	−0.017 (2)	0.057 (3)
C16	0.076 (2)	0.113 (3)	0.103 (3)	0.025 (2)	−0.004 (2)	0.027 (2)
C17	0.0665 (18)	0.079 (2)	0.0737 (19)	0.0133 (17)	−0.0027 (15)	0.0094 (17)

### Geometric parameters (Å, °)

**Table:**

O1—C1	1.198 (3)	S8—C7	1.848 (3)
O2—C3	1.213 (3)	C9—C10	1.511 (4)
C1—C7A	1.506 (3)	C9—H9A	0.9700
N2—C1	1.401 (3)	C9—H9B	0.9700
N2—C3	1.380 (3)	C10—C11	1.500 (6)
N2—C12	1.435 (3)	C10—H10A	0.9700
C3A—C3	1.500 (3)	C10—H10B	0.9700
C3A—C4	1.553 (3)	C11—H11A	0.9600
C3A—C7A	1.529 (3)	C11—H11B	0.9600
C3A—H3A	0.9800	C11—H11C	0.9600
C4—C5	1.504 (4)	C12—C13	1.356 (4)
C4—C9	1.511 (4)	C12—C17	1.375 (4)
C5—H5	0.95 (3)	C13—C14	1.406 (5)
C6—C5	1.323 (5)	C13—H13	0.9300
C6—H6	0.95 (3)	C14—H14	0.9300
C7—C6	1.482 (4)	C15—C14	1.346 (5)
C7—C7A	1.555 (3)	C15—C16	1.353 (6)
C7—H7	0.9800	C15—H15	0.9300
C7A—H7A	0.9800	C16—H16	0.9300
S8—O3	1.4836 (18)	C17—C16	1.387 (4)
S8—C4	1.871 (3)	C17—H17	0.9300
			
O1—C1—N2	124.2 (2)	O3—S8—C4	108.56 (11)
O1—C1—C7A	128.2 (2)	O3—S8—C7	110.62 (11)
N2—C1—C7A	107.5 (2)	C7—S8—C4	80.60 (11)
C1—N2—C12	123.8 (2)	C4—C9—H9A	108.2
C3—N2—C1	113.2 (2)	C4—C9—H9B	108.2
C3—N2—C12	122.7 (2)	C10—C9—C4	116.4 (3)
O2—C3—N2	123.8 (2)	C10—C9—H9A	108.2
O2—C3—C3A	127.8 (2)	C10—C9—H9B	108.2
N2—C3—C3A	108.4 (2)	H9A—C9—H9B	107.3
C3—C3A—C4	112.2 (2)	C9—C10—H10A	108.9
C3—C3A—C7A	105.22 (19)	C9—C10—H10B	108.9
C3—C3A—H3A	110.8	C11—C10—C9	113.2 (3)
C4—C3A—H3A	110.8	C11—C10—H10A	108.9
C7A—C3A—C4	106.71 (19)	C11—C10—H10B	108.9
C7A—C3A—H3A	110.8	H10A—C10—H10B	107.7
C3A—C4—S8	100.79 (16)	C10—C11—H11A	109.5
C5—C4—S8	96.01 (18)	C10—C11—H11B	109.5
C5—C4—C3A	109.5 (2)	C10—C11—H11C	109.5
C5—C4—C9	118.7 (2)	H11A—C11—H11B	109.5
C9—C4—S8	114.65 (19)	H11A—C11—H11C	109.5
C9—C4—C3A	114.4 (2)	H11B—C11—H11C	109.5
C4—C5—H5	124.0 (16)	C13—C12—N2	120.0 (3)
C6—C5—C4	111.4 (3)	C13—C12—C17	121.3 (3)
C6—C5—H5	124.7 (15)	C17—C12—N2	118.7 (2)
C5—C6—C7	111.1 (3)	C12—C13—C14	118.2 (3)
C5—C6—H6	129.2 (19)	C12—C13—H13	120.9
C7—C6—H6	119.5 (19)	C14—C13—H13	120.9
S8—C7—H7	115.3	C13—C14—H14	119.8
C6—C7—S8	97.05 (19)	C15—C14—C13	120.4 (4)
C6—C7—C7A	110.0 (2)	C15—C14—H14	119.8
C6—C7—H7	115.3	C14—C15—C16	121.2 (4)
C7A—C7—S8	101.92 (16)	C14—C15—H15	119.4
C7A—C7—H7	115.3	C16—C15—H15	119.4
C1—C7A—C3A	105.4 (2)	C15—C16—C17	119.6 (4)
C1—C7A—C7	112.6 (2)	C15—C16—H16	120.2
C1—C7A—H7A	110.9	C17—C16—H16	120.2
C3A—C7A—C7	106.0 (2)	C12—C17—C16	119.3 (3)
C3A—C7A—H7A	110.9	C12—C17—H17	120.3
C7—C7A—H7A	110.9	C16—C17—H17	120.3
			
O1—C1—C7A—C3A	−179.5 (3)	C3A—C4—C5—C6	−62.6 (3)
O1—C1—C7A—C7	65.4 (4)	C9—C4—C5—C6	163.5 (3)
N2—C1—C7A—C3A	2.2 (3)	S8—C4—C9—C10	65.0 (3)
N2—C1—C7A—C7	−112.9 (2)	C3A—C4—C9—C10	−179.3 (3)
C3—N2—C1—O1	176.9 (3)	C5—C4—C9—C10	−47.5 (4)
C3—N2—C1—C7A	−4.8 (3)	C7—C6—C5—C4	0.7 (3)
C12—N2—C1—O1	3.5 (4)	S8—C7—C6—C5	−42.9 (3)
C12—N2—C1—C7A	−178.1 (2)	C7A—C7—C6—C5	62.5 (3)
C1—N2—C3—O2	−175.5 (2)	S8—C7—C7A—C1	156.02 (18)
C1—N2—C3—C3A	5.3 (3)	S8—C7—C7A—C3A	41.3 (2)
C12—N2—C3—O2	−2.1 (4)	C6—C7—C7A—C1	53.9 (3)
C12—N2—C3—C3A	178.8 (2)	C6—C7—C7A—C3A	−60.8 (3)
C1—N2—C12—C13	−100.8 (3)	O3—S8—C4—C3A	−51.85 (18)
C1—N2—C12—C17	79.6 (3)	O3—S8—C4—C5	−163.06 (17)
C3—N2—C12—C13	86.5 (3)	O3—S8—C4—C9	71.5 (2)
C3—N2—C12—C17	−93.1 (3)	C7—S8—C4—C3A	56.87 (16)
C4—C3A—C3—O2	−67.0 (3)	C7—S8—C4—C5	−54.33 (18)
C4—C3A—C3—N2	112.1 (2)	C7—S8—C4—C9	−179.8 (2)
C7A—C3A—C3—O2	177.3 (2)	O3—S8—C7—C6	161.81 (16)
C7A—C3A—C3—N2	−3.5 (3)	C4—S8—C7—C6	55.41 (17)
C3—C3A—C4—S8	−156.84 (17)	O3—S8—C7—C7A	49.62 (19)
C3—C3A—C4—C5	−56.4 (3)	C4—S8—C7—C7A	−56.78 (16)
C3—C3A—C4—C9	79.6 (3)	C4—C9—C10—C11	−176.4 (4)
C7A—C3A—C4—S8	−42.1 (2)	N2—C12—C13—C14	−178.7 (3)
C7A—C3A—C4—C5	58.3 (3)	C17—C12—C13—C14	0.9 (4)
C7A—C3A—C4—C9	−165.7 (2)	N2—C12—C17—C16	178.1 (3)
C3—C3A—C7A—C1	0.7 (3)	C13—C12—C17—C16	−1.5 (4)
C3—C3A—C7A—C7	120.3 (2)	C12—C13—C14—C15	0.0 (5)
C4—C3A—C7A—C1	−118.6 (2)	C16—C15—C14—C13	−0.2 (6)
C4—C3A—C7A—C7	1.0 (3)	C14—C15—C16—C17	−0.4 (6)
S8—C4—C5—C6	41.1 (3)	C12—C17—C16—C15	1.3 (5)

### Hydrogen-bond geometry (Å, °)

**Table:**

Cg1 is the centroid of the C12–C17 phenyl ring.

**Table:**

*D*—H···*A*	*D*—H	H···*A*	*D*···*A*	*D*—H···*A*
C5—H5···O1^i^	0.95 (2)	2.51 (3)	3.326 (4)	144 (2)
C17—H17···O3^ii^	0.93	2.57	3.315 (3)	137
C7—H7···Cg1^iii^	0.98	2.77	3.693 (3)	157

Symmetry codes: (i) −*x*, *y*+1/2, −*z*+1/2; (ii) *x*+1/2, −*y*+1/2, −*z*; (iii) *x*−1, *y*, *z*.
